# The 3D Modeling System for Bioaerosol Distribution Based on Planar Laser-Induced Fluorescence

**DOI:** 10.3390/s21082607

**Published:** 2021-04-08

**Authors:** Siying Chen, Yuanyuan Chen, Yinchao Zhang, Pan Guo, He Chen, Huiyun Wu

**Affiliations:** 1 School of Optics and Photonics, Beijing Institute of Technology, Beijing 100081, China; csy@bit.edu.cn (S.C.); 3120160297@bit.edu.cn (Y.C.); ychang@bit.edu.cn (Y.Z.); guopan@bit.edu.cn (P.G.); 2 Academy of Military Medical Sciences, Academy of Military Sciences, Beijing 100850, China

**Keywords:** bioaerosol, PLIF, concentration, 3D, reconstruction

## Abstract

Although it is quite challenging to image and analyze the spatial distribution of bioaerosols in a confined space, a three-dimensional (3D) modeling system based on the planar laser-induced fluorescence (PLIF) technique is proposed in this paper, which is designed to analyze the temporal and spatial variations of bioaerosol particles in a confined chamber. The system employs a continuous planar laser source to excite the fluoresce, and a scientific complementary metal oxide semiconductor (sCMOS) camera to capture images of 2048 × 2048 pixels at a frame rate of 12 Hz. While a sliding platform is moving back and forth on the track, a set of images are captured at different positions for 3D reconstruction. In this system, the 3D reconstruction is limited to a maximum measurement volume of about 50 cm × 29.7 cm × 42 cm, with a spatial resolution of about 0.58 mm × 0.82 mm × 8.33 mm, and a temporal resolution of 5 s. Experiments were carried out to detect the PLIF signals from fluorescein aerosols in the chamber, and then 3D reconstruction was used to visualize and analyze the diffusion of aerosol particles. The results prove that the system can be applied to clearly reconstruct the 3D distribution and record the diffusion process of aerosol particles in a confined space.

## 1. Introduction

Bioaerosols usually refer to the aerosols containing biological particles such as bacteria, viruses, and pollen, which are strongly associated with human lives [1,2]. The leakage and spread of infectious or allergenic biological aerosols may significantly infect the human respiratory system, and even cause nerve damage [3]. Now, increasing attention has been directed toward the effects of indoor fungal bioaerosol exposure on health and safety, especially in an enclosed space [4]. Generally, the factors, such as airflows and walking activities of indoor occupants, may affect the variation of fungal and bacterial bioaerosols in indoor environments [5]. Therefore, it is of great significance to measure and analyze the distribution of biological aerosols in a confined environment. The laser-induced fluorescence (LIF) is a highly sensitive technique that can discriminate between different biological particles and non-biological particles and has been applied for effective detection and characterization of bioaerosols [6,7,8].

The LIF-based standoff detection on bioaerosols has been widely applied [9,10,11]. Based on the fluorescence images of different spectral bands, the LIF system can identify bacteria contamination on the target object [12]. Meanwhile, several approaches have been introduced to optimize the three-dimensional (3D) imaging system based on planar LIF (PLIF) and volumetric LIF (VLIF) techniques. For example, Cho et al. developed a scanned PLIF system to detect the relative concentration of OH in multiphase combustion flow fields [13]. By scanning the laser sheet across different spatial locations, multiple images for 3D imaging can be effectively captured with the PLIF technique. Miller et al. presented a 3D PLIF imaging system using toluene as the tracer and visualized the co-flow jet mixing with ambient air [14]. Instead of scanning the laser sheet, the VLIF system can capture the volumetric fluorescence from different angles using multiple cameras, with a higher temporal and spatial resolution [15,16]. The VLIF technique has also been applied to image 3D concentration fields in the turbulent gaseous free jet using four complementary metal oxide semiconductor (CMOS) cameras [17]. Furthermore, Li et al. have studied the reconstruction of 3D flame structures using VLIF signals from eight camera views [18].

Conventional LIF technique generally detects biological particles along one line or at a specific position in the measurement environment [9,10,11,12,19]. Accordingly, only limited details could be captured from fluorescence signals for analyzing the distribution of biological aerosols. With a high temporal and spatial resolution, the 3D imaging system based on PLIF or VLIF techniques can be used to analyze the spatial distribution and dynamic process of the target in a non-intrusive manner. This system has been applied extensively for studies in combustion diagnosis, jet flows, and catalytic reactions [20,21,22]. However, the 3D LIF measurement generally requires the scanned laser sheet or multiple cameras [23,24], which is optically complex and less flexible. Additionally, the measurement volume of a 3D imaging system is limited and normally does not exceed 50 mm × 50 mm × 50 mm. Therefore, few studies have been reported on applying the PLIF or VIF techniques for 3D imaging of bioaerosols in a larger space.

In this study, we designed and built a 3D modeling system to capture images of fluorescence intensity and visualize the spatial distribution of bioaerosols using the PLIF technique. Different from the multi-camera detection system, this system applies a planar laser beam from the continuous-wave (CW) laser to excite the target particles, and a scientific CMOS (sCMOS) camera to capture fluorescence images. The methods of image denoising, geometric correction, and 3D reconstruction are employed to reconstruct the 3D distribution of the target particles in a 500 mm × 500 mm × 1000 mm chamber. It was found that a new approach can be applied to achieve 3D imaging of fluorescein aerosols with sufficient temporal and spatial resolution in a larger volume. Therefore, the paper presents and discusses the feasibility of using the PLIF technique to achieve 3D imaging of the relative concentration of bioaerosols in the enclosed environment.

## 2. System and Experiment

### 2.1. Structure and Equipment

**Figure 1:**
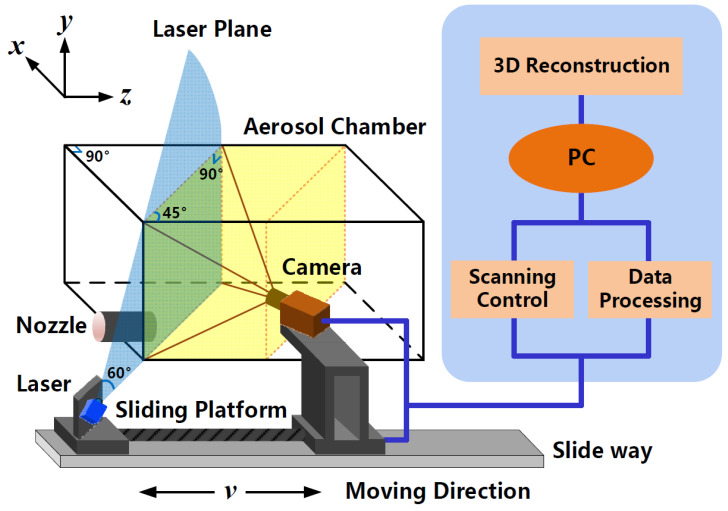
Schematic of the 3D modeling system.

As shown in Figure 1, the schematic of the 3D modeling system is presented. With a wavelength of 450 nm and laser power of 100 mW, a beam-shaping device is equipped on the CW laser to form a laser sheet at an angle of 60°. The detection plane forms an angle of 45° with the Y-Z plane. The sCMOS camera (HAMAMATSU ORCA-Flash4.0 V3, Hamamatsu, Japan) with an optical lens (Kowa LM16XC) is employed to capture PLIF images of 2048 × 2048 pixels, with each pixel size of 6.5 μm × 6.5 μm. The aerosol chamber is made of quartz glass, with a volume of 500 mm × 500 mm × 1000 mm. The bandpass filter (Edmund #33-331, Barrington, New Jersey, USA) is placed in front of the lens to select the specific fluorescence signals in the wavelength range from 500 nm to 600 nm. The laser and camera are fixed on a sliding platform, with the imaging orientation perpendicular to the laser plane. Driven by a motor, the sliding platform can move back and forth along the Z-axis. The main parameters of the 3D modeling system are listed in Table 1.

### 2.2. Experimental Design

With a high quantum yield, the fluorescein (C_20_H_12_O_5_) was employed as the reagent for fluorescence excitation in the experiments. The yielded fluorescence signals peaked at the wavelength of approximately 510 nm, with the excitation laser of 450 nm [25].

In the experiments, fluorescein solution was atomized by a nebulizer at an atomization rate of 0.5 mL/s to generate test aerosols with the mass median diameter of 3.9 μm, and aerosol particles with particle size smaller than 5 μm exceed 65%. The resulting particles were filled into the aerosol chamber through a nozzle. When the laser excited the fluorescein, PLIF images were captured by the camera with the frame rate of 12 Hz. The indoor lights were switched off during the experiments.

The following experiments were carried out to validate the system functions. In the first step, when the fluorescein solution at a concentration of 0.1 g/L was atomized into the chamber, the two-dimensional (2D) PLIF images were captured by the camera and went through the image denoising, geometric correction, and coordinate transformation. Then, the fluorescein solutions at concentrations of 0.05 g/L and 0.1 g/L were atomized into the chamber separately, 2D PLIF images were continuously collected at the same position to compare the changes of the 2D distribution of fluorescein solution aerosols at different concentrations. Finally, we atomized 0.1 g/L fluorescein solution, and collected PLIF images at different positions along the Z-axis for 30 s, while the sliding platform was moving at a speed of 10 cm/s. And the captured images were used to study the 3D reconstruction of relative concentrations of aerosols, and the 3D variation process of the aerosols.

## 3. Data Processing

### 3.1. Image Denoising

In order to increase the signal-to-noise ratio (SNR) and reduce the stray light during the process of collecting PLIF images, the background subtraction and pixel binning methods were applied for image denoising. The original PLIF images captured at the resolution of 2048 × 2048 pixels were resized to 512 × 512 pixels by means of a 4 × 4 pixel binning process. The imaging plane covered an area of 42 cm × 42 cm in the camera. Therefore, the resolution of the 2D image was about 0.82 mm × 0.82 mm. Before aerosols entered the chamber, the background noises were collected when the laser was switched on. Background subtraction was performed by subtracting the noise image from the original PLIF images. Additionally, the wavelet thresholding technique was applied for image denoising. The soft threshold function can be written as [26]:

(1) $$w_{\delta }=\{\begin{array}{c} \mathrm{sgn}(w)\;(|w|-\delta ),\;\;\;|w|\;\geq \delta \\ \;\;\;\;\;\;\;\;\;\;\;\;0,\;\;\;\;\;\;\;\;\;\;\;|w|\;<\delta \end{array}$$

where w is the coefficient vector by wavelet decomposition of the image, δ is the threshold, which can be calculated as [27]:

(2) $$\delta =\{\begin{array}{c} \sigma \left[0.3936+0.1829\left(\frac{\ln N}{\ln2}\right)\right],\;\;\;N>32\\ \;\;\;\;\;\;\;\;\;\;\;\;\;\;\;\;\;\;0,\;\;\;\;\;\;\;\;\;\;\;\;\;\;\;\;\;N\leq 32 \end{array}$$

where N is the length of the signal vector, σ represents the variance of noise, which can be written as [27]:

(3) $$\sigma =median|f_{i}|/0.6745$$

where $f_{i}$ is the wavelet coefficient vector at unit scale.

### 3.2. Geometric Correction of Laser Intensity

**Figure 2:**
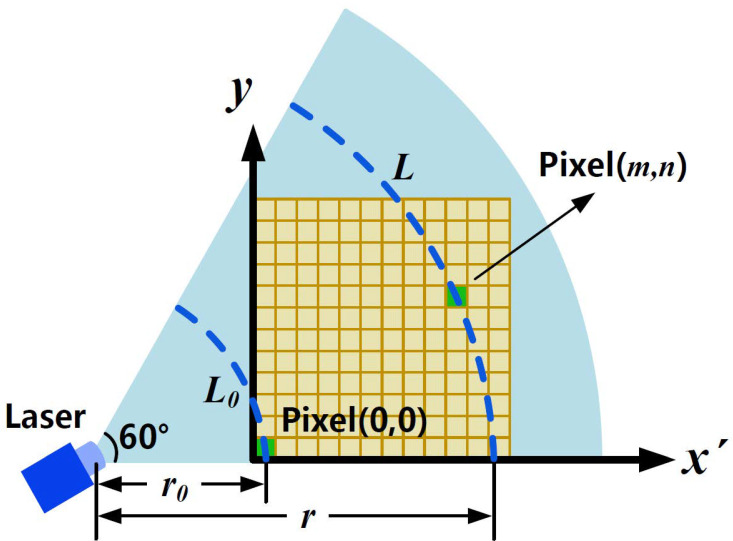
Schematic of laser detection plane.

As shown in Figure 2, the laser source is a 60° fan-shaped beam, the laser energy in each pixel of the detection plane is different and should be corrected. Without considering the influence of aerosol particles, the geometric correction of the laser intensity is discussed and analyzed below.

r is defined as the detection distance of the pixel (m,n), L is defined as the arc length of the laser plane with the detection distance of r. $r_{0}$ is the detection distance of pixel (0,0), $L_{0}$ is defined as the arc length with the detection distance of $r_{0}$. The ratio of laser intensity $I_{0}$ at the location of the pixel (0,0) and laser intensity I(m,n) can be expressed as:

(4) $$\frac{I_{0}}{I\left(m,n\right)}=\frac{L(m,n)}{L_{0}}=\frac{r\left(m,n\right)}{r_{0}}=\frac{\sqrt{{\left(r_{0}+m\right)}^{2}+n^{2}}}{r_{0}}$$

Thus, the geometric correction ratio is described as:

(5) $$P\left(m,n\right)=\frac{I_{0}}{I\left(m,n\right)}=\frac{r_{0}}{\sqrt{{\left(r_{0}+m\right)}^{2}+n^{2}}}$$

**Figure 3:**
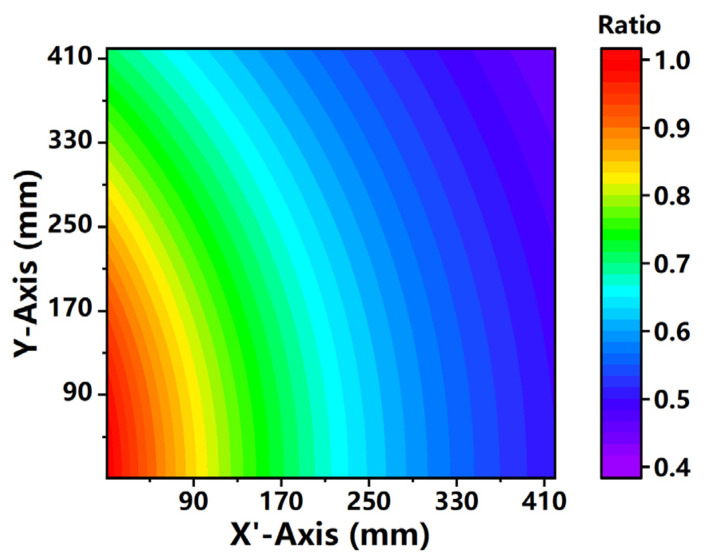
The ratios of laser intensities (the imaging plane is 42 cm × 42 cm).

As shown in Figure 3, the ratio of laser intensity at each pixel is related to the detection distance. It is obvious that the laser intensity gradually gets weakened from the lower-left corner to the upper right corner. The uneven distribution of laser intensity imposes a significant effect on PLIF signals. Therefore, each pixel intensity of PLIF images should be a multiple of the geometric correction ratio to get the accurate fluorescence signal.

### 3.3. 3D Reconstruction

**Figure 4:**
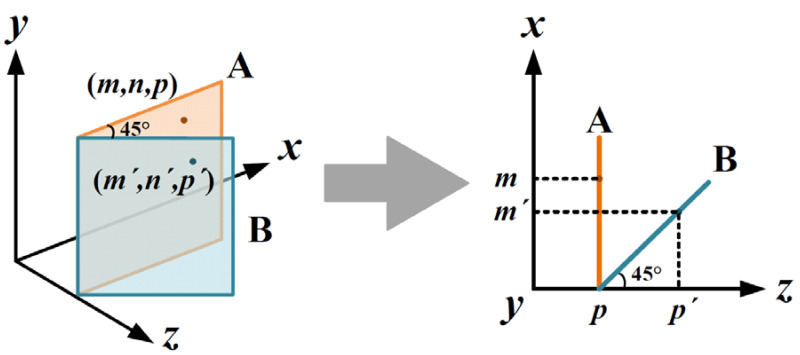
Schematic of coordinate transformation.Therefore, a fundamental step in the 3D reconstruction is to realize the spatial coordinate transformation from image A to image B.

The pixels of PLIF images are identified by 3D spatial coordinates and the position of the pixel (0,0,p) in each PLIF image is recorded according to the position of the sliding platform. As shown in Figure 4, the original 3D spatial coordinates of each pixel are recorded as (m,n,p) in image A. However, the laser plane forms an angle of 45° with the chamber sidewall. In order to demonstrate the right 3D reconstruction, the spatial position of the pixel (m,n,p) should be corrected as $\left(m^{\prime },n^{\prime },p^{\prime }\right)$ in image B.

(6) $$\begin{array}{c} m^{\prime }=\frac{m}{\sqrt{2}}\\ n^{\prime }=n\\ p^{\prime }=p+\frac{m}{\sqrt{2}} \end{array}$$

## 4. Result and Discussion

### 4.1. Analysis of Fluorescence Attenuation

**Figure 5:**
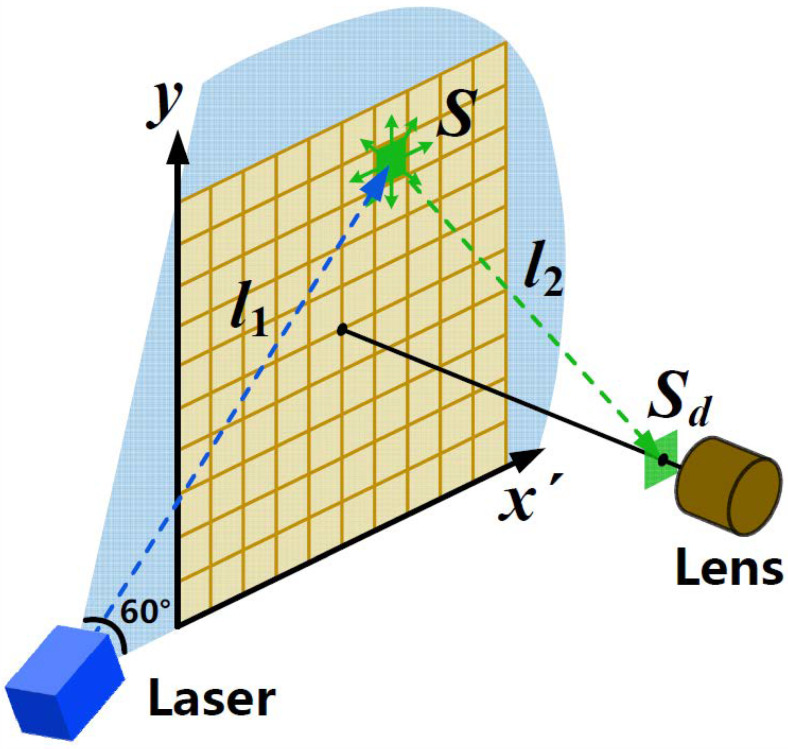
Schematic of fluorescence attenuation.

As shown in Figure 5, the light is emitted from the laser source to the target point S, and then the fluorescence light excited from the point S enters the sCMOS camera. Both laser light and fluorescence light will be attenuated along the path by the aerosols in the chamber. We define S as the intensity of the excited fluorescence, without considering aerosol attenuation. Therefore, the ratio of detected fluorescence intensity $S_{d}$ to S can be approximately expressed as [28]:

(7) $$\frac{S_{d}}{S}=10^{-(A_{ex}+A_{em})/2}$$

where $A_{ex}$ is the absorbance at the excitation wavelength, $A_{em}$ represents the absorbance of fluorescence.

According to Lambert-Beer’s law, the absorbance can be obtained as below [29]:

(8) $$A_{ex}+A_{em}=\varepsilon_{1}\int_{0}^{l_{1}}C_{1}(l)dl+\varepsilon_{2}\int_{0}^{l_{2}}C_{2}(l)dl$$

where $\varepsilon_{1}$ and $\varepsilon_{2}$ are the extinction coefficients for the excitation and emission wavelengths respectively, $l_{1}$ is the optical path of laser light, $l_{2}$ is the optical path of fluorescence, $C_{1}\left(l\right)$ and $C_{2}\left(l\right)$ are the local concentrations of aerosols.

**Figure 6:**
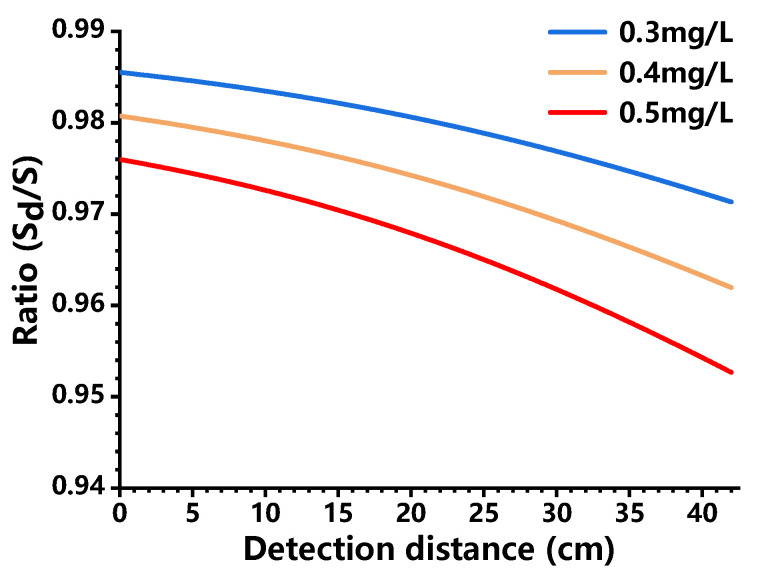
Ratio as a function of detection distance at three different concentrations (0.3 mg/L, 0.4 mg/L, 0.5 mg/L).

According to Equation (8) and Figure 5, the detected fluorescence intensity $S_{d}$ at a specific pixel depends on the concentration distribution of fluorescein in the chamber, the position of the conjugated object point, and extinction coefficients $\left(\varepsilon_{1},\varepsilon_{2}\right)$ which are considered as constants with negligible changes. In the experiments, it was observed that the fluorescein solution samples exhibited a maximum concentration of 0.1 g/L, with an atomization rate of 0.5 mL/s. As mentioned above, the aerosol chamber has a volume of 250 L, and the atomization process took place within 50 s, so the average concentration peaked at 0.01 mg/L. Because the fluorescein characterized the non-uniform concentration distribution in the chamber, we assumed that the concentration at a certain location is dozens of tens times higher than the average value. When the concentration was 30, 40, and 50 times higher than the average value, the ratio $\left(S_{d}/S\right)$ was calculated according to Equation (7), with the results shown in Figure 6. It was found that the ratio decreased with the increasing detection distance, showing a downward trend. The light attenuation rate at a certain concentration could be obtained by subtracting the ratio from 1, which was 2.87% (0.3 mg/L), 3.80% (0.4 mg/L) and 4.73% (0.5 mg/L), respectively. It proves that the light attenuation due to aerosols did not exceed 5% even when the concentration reached 0.5 mg/L which was 50 times higher than the average value. Therefore, we analyzed the distribution of relative concentrations based on the detected fluorescence intensity in the following data processing, without considering the light attenuation.

### 4.2. Analysis of Image Processing

**Figure 7:**
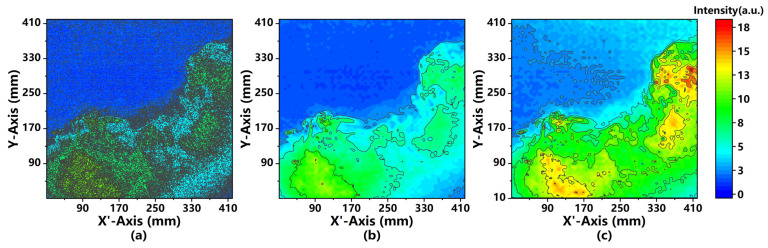
The 2D intensity distribution of planar laser-induced fluorescence (PLIF) images captured at the 20th second. The signals in (**a**) were processed with background subtraction and pixel merging, the signals in (**b**) were processed with background subtraction, pixel merging, and wavelet denoising, and the signals in (**c**) were further processed with geometric correction, in addition to the above mentioned three methods. The concentration of the atomized fluorescein solution was 0.1 g/L, and the signals were obtained with the laser power of 100 mW, a 25 ms frame, and an atomization rate of 0.5 mL/s.

To collect the PLIF images, we prepared a fluorescein solution at the concentration of 0.1 g/L, which was atomized by a nebulizer into the chamber with an atomization rate of 0.5 mL/s. The imaging system continuously captured the PLIF images in the atomization process. Figure 7 shows the PLIF images captured at the 20th second in different pre-processing stages. As shown in Figure 7a, it’s hard to distinguish the intensity distribution through PLIF images processed with background subtraction and pixel binning methods. However, the smoothed PLIF images which went through background subtraction, pixel merging, and wavelet denoising, present a more clear and virtualized intensity distribution, as shown in Figure 7b. In addition to the image processing methods used in Figure 7a,b, a geometric correction was applied to analyze the laser intensity, and it’s found that the fluorescence intensity of images is significantly improved and the difference of PLIF signal intensities is more obvious, as shown in Figure 7c. Therefore, according to the comparative results, the distribution and diffusion of particles can be further recorded and analyzed.

### 4.3. Comparison of 2D Intensity Distributions

**Figure 8:**
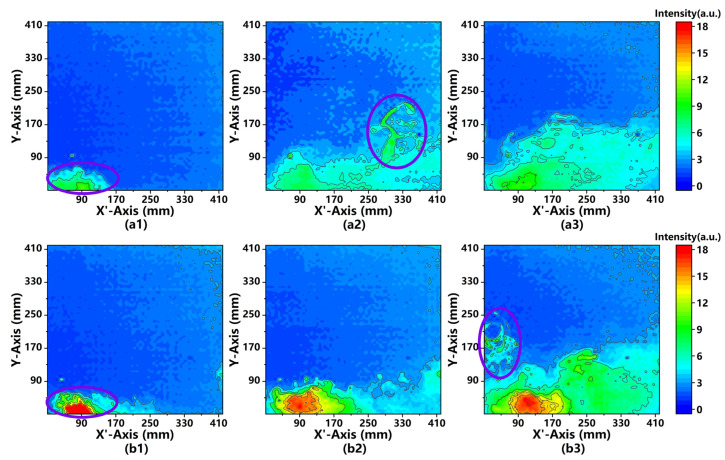
The 2D intensity distribution of PLIF images captured at the 2.5th, 7.5th, and 12.5th second. The concentration of the atomized fluorescein solution in (**a1**), (**a2**) and (**a3**) is 0.05 g/L, and the concentration of the atomized fluorescein solution in (**b1**), (**b2**) and (**b3**) is 0.1 g/L. The signals were obtained with the laser power of 100 mW, a 25 ms frame, and an atomization rate of 0.5 mL/s.

To assess the system performance of detecting the 2D distributions of aerosols, we selected the fluorescein solutions at two different concentrations for comparison. In the experiments, fluorescein solutions were atomized by a nebulizer with an atomization rate of 0.5 mL/s. As shown in Figure 8, the PLIF images at the 2.5th, 7.5th, and 12.5th seconds were collected while the sliding platform remained stationary. The fluorescein solutions at a concentration of 0.05 g/L were prepared for atomization in Figure 8a1–a3, and 0.1 g/L solutions were prepared in Figure 8b1–b3.

As outlined in purple in Figure 8a1,b1, it can be observed that PLIF signals present a high intensity at the 2.5th second. This is because the nozzle was placed in the lower part of the chamber. PLIF signals were getting stronger with the injection of particles. Figure 8a2,b2 exhibit the 2D distribution of particles at the 7.5th second. A significant increase in signals can be observed at the bottom of the entire cross-sectional area, indicating that the fluorescein aerosols first diffused across the lower part of the chamber. In addition, as outlined in purple in Figure 8a2,b3, the unusual distribution of PLIF signals can be observed, which might result from small-scale turbulence. Figure 8a3,b3 shows that the 2D distribution of upward-diffused particles at the 12.5th second. In addition, the peak intensity of signals is nearly twice as much in Figure 8b1–b3 as in Figure 8a1–a3. According to the PLIF images, the 2D distribution of fluorescein solution particles can clearly reflect the bottom-up diffusion.

### 4.4. Diffusion Process of 3D Distribution

To evaluate the system performance of monitoring 3D distribution and diffusion of aerosols, we collected PLIF images at different positions along the Z-axis during the atomization process. The 0.1 g/L fluorescein solution was atomized by a nebulizer with an atomization rate of 0.5 mL/s in the experiment. While the sliding platform moved back and forth with a speed of 10 cm/s, the camera captured the PLIF images with an exposure time of 25 ms/frame and a frame rate of 12 Hz. The laser plane forms an angle of 45° with the chamber sidewall. Through coordinates transformation of image pixels, the 3D distributions of fluorescein solution particles were reconstructed. The 60 PLIF images were taken to reconstruct a 3D distribution at a temporal resolution of 5 s and a spatial resolution of about 0.58 mm × 0.82 mm × 8.33 mm.

**Figure 9:**
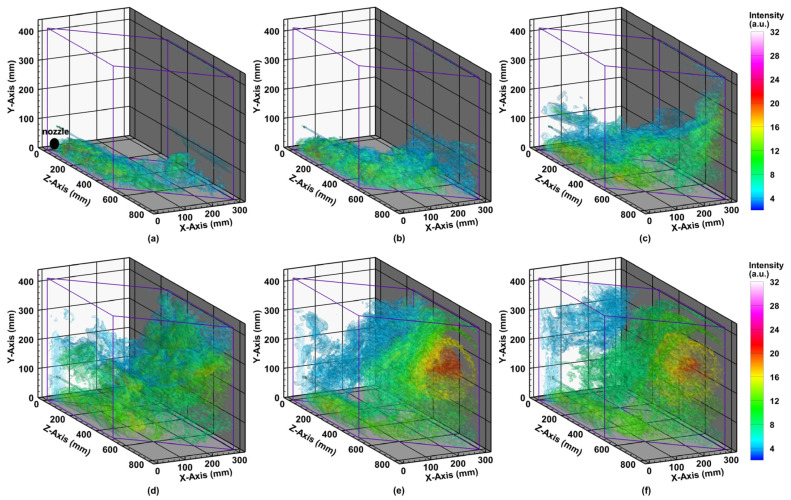
The 3D intensity distribution of PLIF images in 0–5 s (**a**), 5–10 s (**b**), 10–15 s (**c**), 15–20 s (**d**), 20–25 s (**e**) and 25–30 s (**f**). The concentration of the atomized fluorescein solution is 0.1 g/L, and the moving speed of the sliding platform is 10 cm/s. The signals were obtained with the laser power of 100 mW, a 25 ms frame, and an atomization rate of 0.5 mL/s.

Figure 9 shows the 3D distributions of particles within 30 s when fluorescein was injected into the chamber. Figure 9a,b presents the initial diffusion process of the fluorescein solution aerosols in the chamber in the first 10 s, and Figure 9a marks out the position of the inlet nozzle. It can be observed in Figure 9a,b that the aerosols are spreading in the bottom and tend to rise up along the wall. As shown in Figure 9c,d, it can be observed that the density of aerosols at the bottom area becomes stronger, and aerosols keep rising from 10th to 20th second. Lastly, according to Figure 9e,f, the fluorescence signals get much stronger during the atomization process. The turbulence is clearly found in the upper area of the chamber. In the entire diffusion process, it took about 20–30 s for the chamber to be filled with fluorescein solution aerosols, which was quite slow. Therefore, the 3D imaging temporal resolution of 5 s could still have useful value.

If more complicated data processing methods such as interpolation and curve fitting are applied for this system, it’s feasible to demonstrate the animation effects of the variation of 3D distribution, which can promote further studies on the dynamics of biological aerosols.

## 5. Conclusions

In this paper, a 3D modeling system was designed and demonstrated to visualize the distribution of relative concentrations of fluorescein particles using the PLIF technique. The laser plane at an angle of 60° was utilized as the excitation source to induce fluorescence, and a sCMOS camera was employed to collect PLIF images. The background subtraction, pixel merging, and wavelet denoising methods were selected for image processing. In the experiments, the system was used to image 2D and 3D distributions of particles, and then the concentration variation of fluorescein in the atomization process was analyzed to verify the reliability and performance of the system. Despite a relatively lower temporal and spatial resolution of the system, the experiment results prove that the system is able to visualize the 3D diffusion process of aerosols in a 500 mm × 500 mm × 1000 mm chamber, which is important for modeling and studying the distribution, leakage, and diffusion of bioaerosols. According to fluorescence spectra of bioaerosol particles, the system can select the specific fluorescence signals for imaging and analysis. Furthermore, by placing simulation models in the chamber, the system can be used to analyze a more realistic diffusion process of particles in various confined environments in future studies.

## Figures and Tables

**Table 1:** The main parameters of the system.

Component	Parameter	Value
Laser	Laser Power	100 mW
	Laser Wavelength	450 nm
Lens	Focal Length	16 mm
	Imaging Distance	30 cm
Camera	Quantum Efficiency	0.82 (500–600 nm)
	Cell Size	6.5 μm × 6.5 μm
	Effective Area	13.3 mm × 13.3 mm

## Data Availability

Not applicable.
